# Prospective study of pharmacokinetics of isavuconazole in critically ill patients undergoing continuous hemodialysis with and without acute-on-chronic liver failure

**DOI:** 10.1016/j.aicoj.2026.100082

**Published:** 2026-05-14

**Authors:** Emily Behrens, Christina König, Dominik Jarczak, Jens Martens-Lobenhoffer, Stefanie M. Bode-Böger, Stefan Kluge, Sebastian G. Wicha, Valentin Fuhrmann, Jörn Grensemann

**Affiliations:** a Department of Clinical Pharmacy, Institute of Pharmacy, University of Hamburg, Bundesstraße 45, 20146 Hamburg, Germany; b Department of Intensive Care Medicine, University Medical Center Hamburg-Eppendorf, Martinistraße 52, 20246 Hamburg, Germany; c Institute of Clinical Pharmacology, Otto-von-Guericke University, Leipziger Str. 44, 39120 Magdeburg, Germany; d Department of Internal Medicine, Cellitinnen Krankenhaus Heilig Geist, Graseggerstrasse 105, 50737 Cologne, Germany

**Keywords:** Isavuconazole, Antifungal, Intensive care, Integrated dialysis pharmacometric model, Continuous hemodialysis, Renal replacement therapy, Acute-on-chronic liver failure, Monte Carlo simulation

## Abstract

**Background:**

Patients with acute-on-chronic liver failure (ACLF) are highly vulnerable to invasive fungal infections often requiring renal replacement therapy (RRT). The pharmacokinetics (PK) of isavuconazole, an antifungal against *Aspergillus spp.* and *Mucorales*, may be altered in ACLF due to effects of altered distribution and clearance. We investigated the impact of ACLF on isavuconazole PK in critically ill patients undergoing continuous RRT due to a paucity of data in this patient population and aimed to inform decisions about dosing.

**Methods:**

This prospective observational cohort study was conducted at the University Medical Center Hamburg-Eppendorf across twelve intensive care units with 140 beds. We included critically ill adults receiving isavuconazole and continuous RRT. Patients <18 years or with extracorporeal circuits other than RRT were excluded. Plasma, ultrafiltrate, and pre-/postfilter blood samples were analyzed using high-performance liquid chromatography. Key areas of interest were the effect of ACLF and continuous RRT on isavuconazole pharmacokinetics, particularly on clearance. Population PK modeling was performed using, inter alia, the integrated dialysis pharmacometric model in NONMEM®. Probability of target attainment (PTA) for established PK/PD thresholds developed in mice (50% or 90% survival) was assessed using Monte Carlo simulations.

**Results:**

Thirteen patients were enrolled, including 4 with ACLF. A two-compartment model best described the data. Median clearance was similar between ACLF (4.2 L/h) and non-liver failure (4.8 L/h) groups (p = 0.92). Dialysis clearance was negligible (<1% of dose). The 50% efficacy target (AUC_0−24h_/MIC = 24.7) was achieved in 12/13 patients (92%), and the 90% target (AUC_0−24h_/MIC = 33.4) in 8/13 patients (62%). Simulations indicated suboptimal PTA at the *Aspergillus fumigatus* breakpoint of 1 mg/L. Considerable interindividual variability on clearance, particularly in ACLF, led to both sub- and supratherapeutic exposures.

**Conclusion:**

ACLF did not significantly alter isavuconazole clearance during RRT. However, high PK variability and frequent subtherapeutic exposures support routine therapeutic drug monitoring in critically ill patients.

## Introduction

Within the group of critically ill patients, patients with acute-on-chronic liver failure (ACLF) are particularly susceptible to infections associated with a high mortality [1,2]. Besides bacterial pathogens, molds and other fungi may play a role [[3], [4], [5], [6]]. Isavuconazole is a treatment option against *Aspergillus spp.* and is also effective against *Mucorales*. According to the product license, no dose adjustments are required for isavuconazole in patients with liver cirrhosis Child-Pugh A and B while no data are available for liver cirrhosis Child-Pugh C [7]. However, in liver cirrhosis Child-Pugh A and B, clearance (CL) was lowered by 40% and 48%, respectively, and exposure defined as the area under the concentration-time-curve (AUC) was markedly higher with 64% and 84%, respectively [8]. In general, the volume of distribution (V) may increase as a result of capillary leak syndrome and ascites, necessitating higher doses of anti-infective drugs [9]. In contrast, elimination may be decreased due to liver failure, and lower doses may be required [10].

Due to these complex alterations in the pharmacokinetics (PK) in critically ill patients, therapeutic drug monitoring (TDM) might help with therapy adjustments, but sufficient initial dosing strategies could prevent inadequate serum values before TDM results are available and the dose being adjusted. However, the Pharmacokinetic/Pharmacodynamic Study Group of the European Society of Intensive Care Medicine currently neither recommends nor discourages TDM for isavuconazole [11].

Our aim was to determine the influence of ACLF on isavuconazole PK, because in patients with liver cirrhosis, concomitant organ failure in ACLF patients might influence PK parameters like V and CL differently than stable liver cirrhosis. As the most severely ill ACLF patients often suffer from acute kidney injury as well [12] and require renal replacement therapy (RRT) [13,14], we studied the PK of isavuconazole in critically ill patients with ACLF requiring RRT and compared this group with critically ill patients on RRT without ACLF to inform recommendations with regard to monitoring and dosing in the context of ACLF.

## Methods

### Ethics

The study was approved by the Ethics Committee of the Hamburg Chamber of Physicians, Germany (Reference: PV5415). Consent was obtained from the patients’ closest relatives or legal representatives. The study was performed in accordance with the ethical standards as laid down in the 1964 Declaration of Helsinki and its later amendments.

### Study design

The study was conducted as a single center open label observational prospective cohort study.

### Setting and population

The study was conducted at the Department of Intensive Care, University Medical Center, Hamburg-Eppendorf across twelve intensive care units (surgical, conservative, and interdisciplinary) with a total of 140 beds. Patients were eligible if they received isavuconazole for clinical indication and required continuous RRT. Patients <18 years or with an extracorporeal circuit other than the RRT were excluded. According to liver function, patients were grouped into patients with ACLF and patients without ACLF (“no liver failure”, NLF).

ACLF was defined according to the definition of the Chronic Liver Failure (CLIF) consortium and the European Association for the Study of the Liver (EASL) practice guideline on ACLF [12,15].

### Medication

Isavuconazole (Cresemba®, Pfizer, New York, NY, USA), was given over 60 min by infusion pump at a dose of 200 mg q8h via a central venous line (short-term infusion) for the first 48 h and then reduced to 200 mg once daily.

### Renal replacement therapy

RRT was performed as continuous veno-venous hemodialysis (CVVHD) or as a postdilution continuous veno-venous hemofiltration (CVVH) as as previously described [16]. Both methods were performed with Multifiltrate pro® dialysis machines using an Ultraflux® AV1000S hollow-fiber hemofilter (Fresenius Medical Care, Bad Homburg, Germany) with a membrane surface area of 1.8 m^2^. For CVVHD, a regional citrate-calcium anticoagulation was used; and the targeted dialysate or replacement fluid dose was 30 mL/kg/h of actual body weight. CVVH was chosen in cases of severe acidosis due to the technically higher possible blood flow. No filter change occurred during the study period.

### Sampling and storage

Ultrafiltrate and pre- and postfilter blood samples were obtained at the following time points: T0 as the baseline before the first monitored infusion, 30 min (Ta), 1 h (T1), 2 h (T2), 6 h (T6), and 12 h (T12) after the start of infusion. Furthermore, we obtained values after 24 h (before and after end of infusion, T24 and T25) and after 48 h (T48 and T49). In cases of CVVH, postfilter samples were obtained from the extracorporeal circuit before the addition of replacement fluid. All samples were centrifuged immediately, and the supernatant stored at −20 °C until analysis.

### Assay

Total isavuconazole concentrations were quantified by high-performance liquid chromatography with fluorescence detection as published previously [17].

### Statistics

Microsoft Excel 2016 (Microsoft Corp., Redmond, WA, USA) was used for data management and the descriptive statistical analysis. The population pharmacokinetic analysis and Monte-Carlo simulation were performed using the non-linear mixed-effects modelling software NONMEM, version 7.5.0 (Icon Development Solutions, Ellicott City, MD, USA), operated using Perl-speaks-NONMEM (PsN, version 5.4.0) [18]. Data visualization was performed in R (version 4.2.1, R Foundation for statistical computing, Vienna, Austria) and RStudio (version 2022.07.0) [2].

### Population pharmacokinetic analysis

To describe the pre-filter plasma concentrations, one-, two- and three-compartment models with linear elimination were evaluated. Interindividual variability between subjects (IIV) and interoccasion variabilities within a subject across dosing occasions (IOV) were tested on all structural PK parameters and both were assumed to be log-normally distributed. Selected patient characteristics (albumin concentrations, bilirubin concentrations and the liver disease status (ACLF/NLF)) were tested as covariates on the PK parameters. To model the residual unexplained variability, additive, proportional and combined error models were evaluated. Additionally, to model pre-filter, post-filter and effluent concentrations simultaneously, the integrated dialysis pharmacometric model (IDP model) was utilized [19]. The IDP model facilitates estimation of the dialysis CL next to the body CL as well as adsorption to the hemofilter cartridge.

For estimation the first-order conditional estimation with interaction (FOCE-I) was used. A drop in objective function value (ΔOFV) of 3.84 was considered statistically significant at alpha of 5% in the likelihood-ratio test for nested models. For non-nested models the Akaike information criterion (AIC) guided the model building process (drop in AIC: ΔAIC). Mean parameter estimates were used to evaluate differences between ACLF and NLF patients in a t-test.

### PK/PD target and probability of target attainment

With the final model AUC_0−24h_/MIC values were calculated for each individual subject in the dataset. Moreover, Monte-Carlo simulations (N = 1000) were performed to evaluate the probability of target attainment (PTA) under steady-state conditions with daily dosing of 200 mg. Two important PK/PD targets for isavuconazole from a murine infection model are reported in the literature: Seyedmousavi et al. reported an AUC_0−24h_/MIC ratio of 24.7 for a 50% survival rate and Buil et al. described an AUC_0−24h_/MIC value of 33.4 for a 90% survival rate [20,21]. AUC_0−24h_/MIC values were calculated for an MIC of 1, which is the European Committee on Antimicrobial Susceptibility Testing (EUCAST) susceptibility breakpoint for *Aspergillus fumigatus*.

## Results

**Fig. 1:**
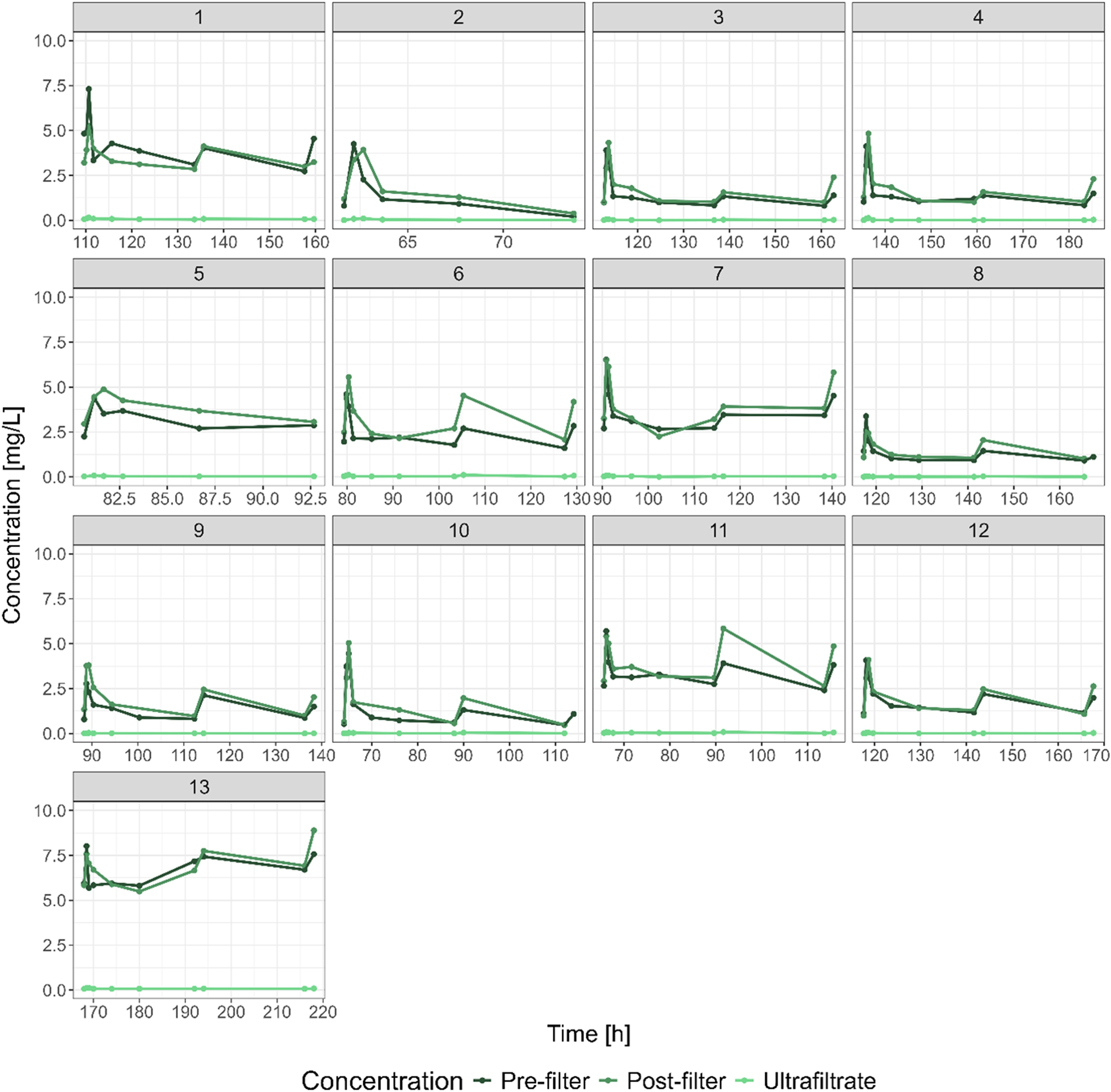
Individual concentration-time curves. No liver failure: patients 1–9, acute-on-chronic liver failure: patients 10–13.

The isavuconazole serum concentration-time data used in this analysis was obtained in 13 critically ill patients on RRT, with 4 of these patients diagnosed with ACLF. A total of 239 serum concentrations and 117 effluent concentrations were available for analysis. Out of the 239 serum concentrations, 122 were pre-filter samples and 117 post-filter samples. Individual concentration-time profiles are shown in Fig. 1. Pre-filter, post-filter and ultrafiltrate concentrations were between 0.2 mg/L – 8.02 mg/L, 0.38 mg/L – 8.89 mg/L, and 0.004 mg/L – 0.161 mg/L, respectively.

An overview of patient characteristics is given in Table 1. All patients were mechanically ventilated and, according to the EASL criteria, all ACLF patients had ACLF grade 3. Individual concentrations are depicted in Fig. 1 as concentration-time curves.

**Table 1:** Clinical characteristics of patients.

	NLF	ACLF
Number of patients	9	4
Males	6 (67%)	3 (75%)
Age (Years)	62 (61; 69)	65 (62; 67)
Weight (kg)	73 (65; 76)	80 (77; 85)
Height (cm)	176 (8)	179 (176; 180)
SAPS II	52 (39; 57)	57 (52; 60)
SOFA	15 (15; 18)	20 (19; 21)
PT (%)	100 (80; 106)	44 (30; 65)
Bilirubin (mg/dL)	1.2 (0.7; 1.4)	11.7 (5.9; 18.4)
Albumin (g/L)	18.3 (16.5; 19.7)	25.6 (19.4; 31.4)
Antithrombin (%)	85 (80; 114)	58 (36; 93)
Child-Pugh	n/a	11 (10; 12)
Grade C	n/a	4 (100%)
CLIF-C ACLF	n/a	64 (62; 67)
CLIF-C AD	n/a	66 (65; 68)
Norepinephrine (μg/kg/min)	0.13 (0.08; 0.26)	0.39 (0.36; 0.42)
Mortality in ICU	4 (44%)	3 (75%)

ACLF: acute-on-chronic liver failure due to liver cirrhosis, NLF: patients without liver failure, PT: prothrombin time, SAPS II: Simplified Acute Physiology Score, SOFA: Sequential Organ Failure Assessment, CLIF-C ACLF: Chronic Liver Failure Consortium Acute-on-chronic liver failure score, CLIF-C AD: Chronic Liver Failure Consortium Acute Decompensation score, Norepinephrine is given as mean dose over the days of measurement, ICU: intensive care unit; n/a: not applicable. Data are given as numbers and percentage or median with quartiles in brackets, as applicable.

### Population pharmacokinetic analysis

A two-compartment model described the pre-filter concentration data best (one-compartment model: ΔAIC +34.468, three-compartment model: ΔAIC +3.761). IIVs on all PK parameters except intercompartmental clearance (Q) led to a statistically significant ΔOFV. The highest ΔOFV was reached when IIV on CL was tested (ΔOFV: −173.35). After including IIV on CL in the model, no additional IIV resulted to be significant (ΔOFV > +0.018). None of the tested covariates and IOVs resulted in a statistically significant ΔOFV (ΔOFV > −3.526). Modeling the pre- and post-filter concentrations simultaneously using the IDP model resulted in the dialysis clearance tending to zero. In line with that, adding the ultrafiltrate concentrations to the dataset revealed that less than 1% of the dose were adsorbed to the hemofilter, indicating that isavuconazole is neither filtrated nor bound to the hemofilter and hemodialysis is not a significant elimination route.

**Fig. 2:**
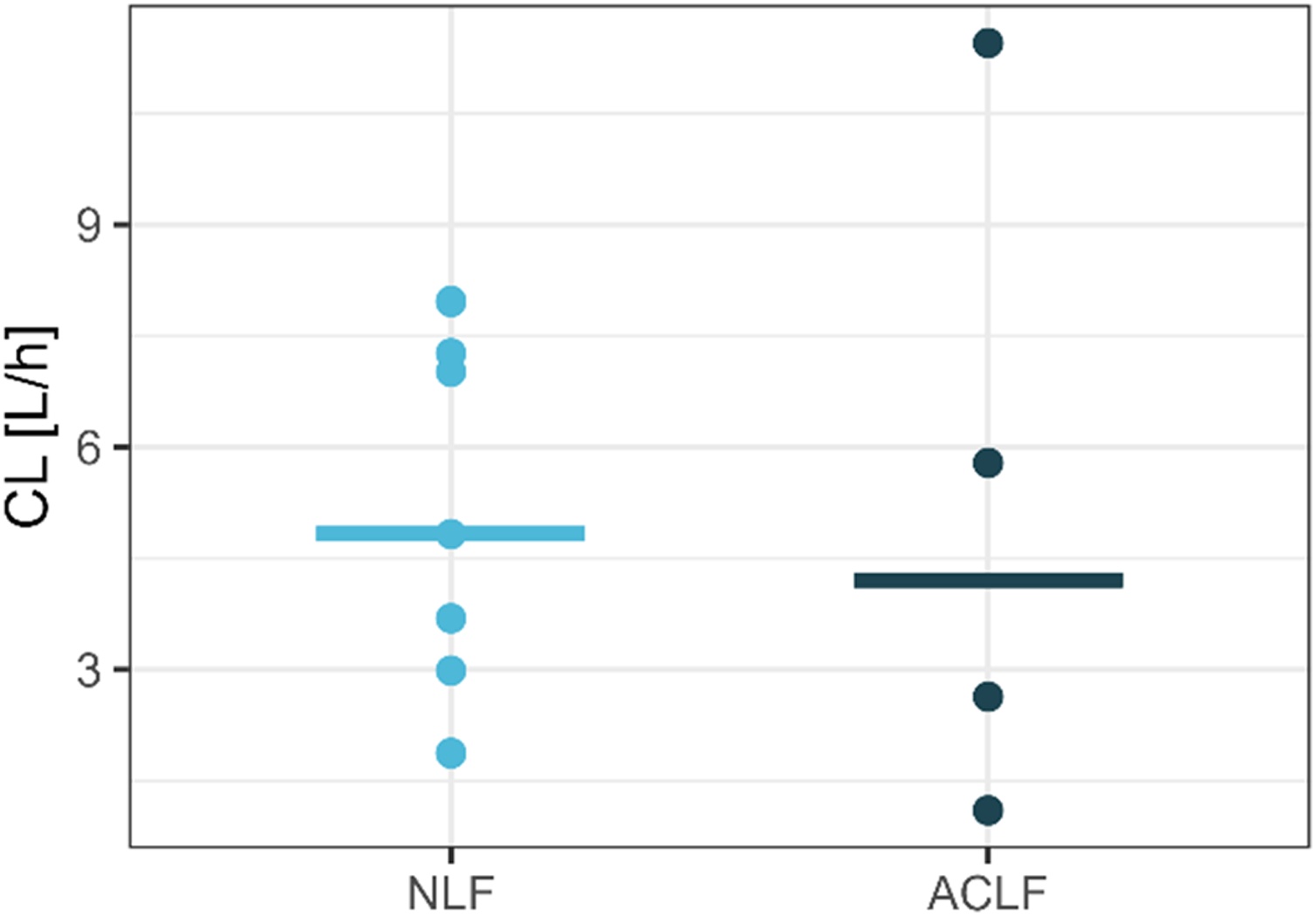
Effect of liver disease status on clearance. dots: geometric means per ID, line: median of subgroup, ACLF: acute-on-chronic liver failure, NLF: no liver failure.

For further analysis, we used the final model of the pre-filter concentrations. Final parameters of that model with relative standard errors are presented in Table 2. In the Supplementary Material, Figure S1 shows the individual pre-filter concentrations and model predictions over time and in Figure S2 the conditional weighted residuals are presented. A prediction corrected visual predictive check can be seen in Figure S3. To further investigate the influence of the liver disease, the geometric means per subject and ACLF/NLF subgroup medians of the individual CL and AUC values are illustrated in Fig. 2. The median value of CL in the ACLF group (4.2 L/h) was smaller in comparison to the median CL of the NLF group (4.8 L/h). In Fig. 2 the ACLF group shows higher variability, but the difference was not statistically significant (t test, two-sided, p = 0.92), thereby confirming the covariate analysis.

**Table 2:** Parameter estimates of final model for pre-filter concentrations.

	Parameter estimates	RSE [%]
CL [L/h]	4.13	25.4
V1 [L]	7.99	13.4
Q [L/h]	91.2	7.4
V2 [L]	293	19.2
IIV on CL [%CV]	78.8	26.5
Prop. error [%CV]	24.3	23.1

CL: clearance, V1: volume of distribution central compartment, Q: intercompartmental clearance, V2: volume of distribution peripheral compartment, IIV: interindividual variability, prop. error: proportional residual error, RSE: relative standard error of the parameter estimate.

### PK/PD target and probability of target attainment

The results of the AUC_0−24h_/MIC calculations are shown in Table 3. In the studied patients, the 50% efficacy target (AUC_0−24h_/MIC = 24.7) was reached in 12/13 patients (92%), and the 90% efficacy target (AUC_0−24h_/MIC = 33.4) in 8/13 patients (62%).

**Table 3:** AUC_0-24h_/MIC and target attainment.

			AUC_0−24h_/MIC target attained	
	ID	AUC/MIC for MIC = 1 mg/L^a^	24.7	33.4
NLF	1	90.0	✓	✓
	2	69.3	✓	✓
	3	27.7	✓	✗
	4	31.0	✓	✗
	5	64.3	✓	✓
	6	51.5	✓	✓
	7	75.8	✓	✓
	8	30.0	✓	✗
	9	29.8	✓	✗
ACLF	10	23.5	✗	✗
	11	72.0	✓	✓
	12	37.5	✓	✓
	13	145.5	✓	✓

AUC: area under curve, MIC: minimal inhibitory concentration, NLF: no liver failure, ACLF: acute-on-chronic liver failure.

a EUCAST susceptibility breakpoint for *Aspergillus fumigatus*.

**Fig. 3:**
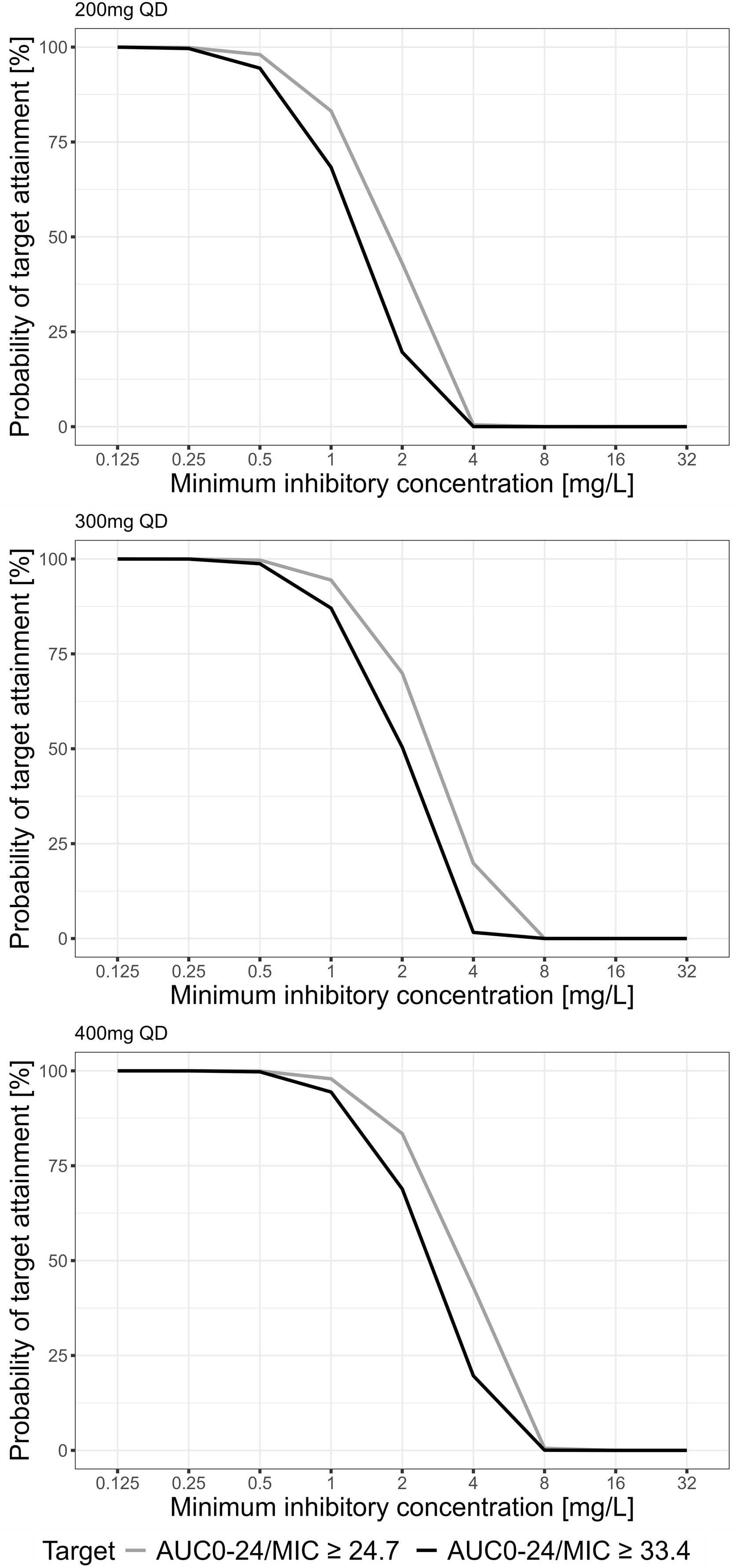
Probability of target attainment for different MICs. Steady-state simulations of a dose of 200 mg, 300 mg, and 400 mg once daily for PK/PD targets of AUC_0−24h_/MIC = 24.7 (murine infection model, 50% survival) and 33.4 (murine infection model, 90% survival).

For the Monte-Carlo-Analysis, the PTA after 1000 simulations for the standard isavuconazole maintenance dose of 200 mg once daily in steady state is illustrated in Fig. 3. At the EUCAST susceptibility breakpoint for *Aspergillus fumigatus* (MIC: 1 mg/L) 83.2% reached the 50% efficacy target (AUC_0−24h_/MIC = 24.7) and 68.3% reached the 90% efficacy target (AUC_0−24h_/MIC = 33.4). Increasing the maintenance dose to 300 mg once daily increased the PTA to 94.4% and 87.0% respectively. A further increase to 400 mg once daily yielded a PTA of 97.9% and 94.4%.

## Discussion

In this study, we developed a population pharmacokinetic (popPK) model of isavuconazole in critically ill patients with and without ACLF undergoing continuous RRT. Patients suffering from concomitant ACLF showed a similar CL compared to patients without liver failure. Most patients (92%, 12/13) achieved the murine 50% efficacy target, and 68% (8/13) of patients the 90% efficacy target. The suboptimal exposures in patients not achieving the targets were linked to lower isavuconazole concentrations in these individuals. Similar proportions for target attainment were also reflected in the PTA simulations.

*In vivo*, isavuconazole is highly protein bound at 98–99% and mainly metabolized by CYP3A4 and possibly by CYP3A5 to a lesser extent; minor pathways include glucuronide and acetylcysteine conjugation, and approximately 33% are excreted unchanged via feces and below 1% in urine [22,23]. No routine TDM is recommended for isavuconazole because data indicate that isavuconazole shows smaller serum concentration variability than voriconazole [24,25]. Voriconazole is primarily metabolized by CYP2C19 with underlying genetic polymorphisms that can lead to variable elimination rates [16]. Furthermore, it has been suggested that isavuconazole has fewer drug-drug interactions mediated by cytochrome enzymes [25]. Nevertheless, plasma concentrations of isavuconazole may be increased or decreased by typical CYP3A4 inhibitors or inducers respectively, and therefore some authors have suggested TDM in cases of hepatic failure, obesity, and fungal strains with high MIC values [26].

Consistent with previous reports, a two-compartment model with first-order elimination best described the data [27]. Our estimated CL was within the same range as previously reported values [28]. The central V in our patient population was approximately at the lower quartile of the previously reported values, and the peripheral V was within the previously reported range, as well [27]. Q was considerably higher than previously described. We think that hypoalbuminemia may play a role, which is often present in critically ill patients, and was also present in our patient cohort [29]. In hypoalbuminemia, the free drug fraction is increased and more likely to be cleared from the central compartment as shown by the higher Q [30]. In our cohort, both groups suffered from hypoalbuminemia and albumin did not prove to be a significant covariate in this regard.

Our model was not significantly influenced by concomitant liver failure. However, the small number of patients may have reduced the power to detect an effect of ACLF on CL or increased the possibility of type II error. Larger studies to further investigate this effect are therefore warranted. Earlier studies i.e. by Schmitt-Hoffmann et al. and Huang et al. observed significant reductions of CL in patients with hepatic impairment, recommending dose reductions in these cases [31,32]. Interestingly, the CL in our study with critically ill patients was higher than in the cohort of Schmitt-Hoffmann et al., who studied healthy individuals and patients with mild and moderate liver failure (Child-Pugh A and B) due to alcoholic cirrhosis [32]. The numerical difference between the estimated CL in our study and the CL reported by Schmitt-Hoffmann et al. might not reflect a biological difference but variability between the different studies, given the large IIV of CL and the residual variability of the popPK model. In our study, the high IIV was particularly evident in the ACLF group with both very low concentrations (below 1 mg/L) and consistently high concentrations (above 5 mg/L) in other subjects. Similar findings were reported by Biagi et al., reinforcing the possibility that TDM could help optimize exposure in this selected patient group [28]. A mathematical model of severe liver failure has previously suggested a dose reduction is required [31]. Interestingly, these authors highlighted that biliary CL may increase to compensate for decreased cytochrome activity in cases of liver failure. However, data actually observing an increase of isavuconazole in the feces are not yet available. In our opinion, a mathematical model focusing only on liver failure cannot predict the complex PK changes present in multi-organ failure as in our study cohort. To describe these dynamic changes (e.g., variable albumin concentrations, changes in cardiac output leading to changes in liver perfusion etc.) accurately, more extensive data like repeated biomarker and/or physiological parameter measurements and modelling of multiple enzymatic elimination pathways would have been required.

No meaningful amounts of isavuconazole were cleared by dialysis, shown by negligible differences between pre- and postfilter concentrations and only minimal ultrafiltrate concentrations. This is consistent with prior studies: Biagi et al. also found a transmembrane CL below 1% [28], as well as Townsend et al. who found no meaningful removal of isavuconazole by dialysis. Interestingly, these authors observed an increase of AUC that was attributed to a heparin-induced displacement of isavuconazole from albumin [33]. Besides the hypoalbuminemia in our patient cohort, this could also explain the slightly higher AUC values in our study. Using the IDP model, we found a reversible adsorption to the filter with less than 1% of the administered dose which has not been previously reported before.

The clinical isavuconazole target attainment highly depends on the MIC of the treated fungus. The isavuconazole breakpoint set by the EUCAST for *Aspergillus fumigatus* is 1 mg/L, however, the wild type MIC distribution also includes 2 mg/L [34]. As of September 2025, the EUCAST lists 592 observations of *Aspergillus fumigatus* isolates with 231 (44%) lower than 1 mg/L, 235 (40%) at 1 mg/L, 41 (7%) at 2 mg/L, and 18 (3%) above 2 mg/L (= resistant). According to our data, a 90% PTA can only be ensured for a MIC of 0.5 mg/L or below, i.e. only 44% of the cases modelled from our patient cohort. At a MIC of 2 mg/L, PTA is below 50%. As the targets were derived from murine studies, they should be considered a “translational” target and are not truly human targets. However, these target values have likewise been used by Desai et al. as reference targets in the evaluation of isavuconazole pharmacokinetics in the SECURE trials [35]. Besides AUC/MIC ratios, a target range of 2.5–5 mg/L has been suggested by Furfaro et al. with the upper limit defined by toxicity effects [36]. This target range was met in only 35% of our pre-filter serum samples with more than 50% of the values below this range, and approximately 11% above. This may be explained by the high IIV in our patient cohort, particularly in ACLF patients. A recent study also found insufficient concentrations of isavuconazole in critically ill patients and the PK/PD model indicated a maintenance dose of 400 mg/d more appropriate to reach adequate target attainment [37]. The safety and tolerability this higher dose has been shown previously [38,39]. In our simulation, an increase of the maintenance dose to 400 mg/d ensured adequate PTA > 90% up to a MIC of 1 mg/L, but still not at a MIC of 2 mg/L which is within the MIC wildtype distribution.

Therefore, we believe that TDM should strongly be considered in critically ill patients irrespective of concomitant liver failure, particularly to prevent from underdosing with the risk of treatment failure because more than 50% of isavuconazole concentrations whilst using standard dosing were too low to achieve sufficient target attainment. In addition, TDM guided dose adjustments would also increase treatment safety in the context of higher maintenance doses and potentially elevated plasma concentrations.

Our study offers several strengths, including the evaluation of a larger patient cohort compared to prior continuous RRT-focused research and the inclusion of individuals with combined hepatic and renal failure. However, the limited sample size remains a limitation, reducing the ability to detect covariate effects. We solely focused on the PK of isavuconazole within a limited time frame of 48 h and do not report clinical outcome measures or toxicity. Therefore, our recommendation to perform TDM is based on simulations and not clinical cure rates. We did not measure the free fraction of isavuconazole that is highly protein bound, however, albumin concentration was no significant covariate in our model. Future research with larger cohorts is needed to clarify the role of liver failure, and to further refine dose adjustment strategies.

## Conclusion

We conducted a prospective, open-label clinical study of isavuconazole in critically ill patients receiving continuous RRT with and without ACLF. Using a pharmacometric analysis, no significant influence of ACLF or continuous RRT on isavuconazole PK, i.e. CL and V, could be demonstrated in our cohort of critically ill patients. Due to the high IIV with both serum concentrations below and above the recommended target range and failure to meet efficacy targets in both groups in our simulations, we recommend routine TDM for isavuconazole in critically ill patients.

## Authors' contributions

EB performed the pharmacokinetic analysis and the Monte-Carlo-Analysis and helped to write the manuscript, CK helped to design the study and helped to write the manuscript, DJ obtained the data and helped with data management, JML and SMBB developed the analytical method and analyzed the samples, SK helped to design the study and to interpret the data and helped to acquire funding, SGW helped to perform the pharmacokinetic analysis and to interpret the data, VF designed the study and interpreted the data and acquired funding, JG designed the study and interpreted the data and wrote the manuscript.

## Consent for publication

Not applicable.

## Ethics approval and consent to participate

The study was approved by the Ethics Committee of the Hamburg Chamber of Physicians, Germany (Reference: PV5415). Consent was obtained from the patients’ closest relatives or legal surrogates. The study was performed in accordance with the ethical standards as laid down in the 1964 Declaration of Helsinki and its later amendments or comparable ethical standards.

## Funding

This study was supported in part by 10.13039/100004319Pfizer (Project-ID: WP2587148). Further expenses were financed by departmental funds.

## Availability of data and material

The data are available from the corresponding author upon reasonable request.

## Declaration of competing interest

CK reports lecture fees from Gilead and Shionogi as well as research support from Infectopharm. SK received research support from Biotest, CytoSorbents, Daiichi Sankyo, Fresenius Medical Care. He also received lecture fees from ADVITOS, CSL Behring, Fresenius Medical Care, Gilead, MSD, Pfizer, Shionogi and consultant fees from ADVITOS, Fresenius, Gilead, MSD and Pfizer. SGW reports grants from Boehringer Ingelheim, consulting fees from Medicines for Malaria Venture, Merck KGaA, UTIlity Therapeutics, and lecture fees from GlaxoSmithKline. JG received research support and lecture fees from Infectopharm outside of the presented work, and research support from Pfizer for the conduct of this study.
