# Supplementary material for: Prospective study of pharmacokinetics of isavuconazole in critically ill patients undergoing continuous hemodialysis with and without acute-on-chronic liver failure
Source: Ann Intensive Care. 2026 May 14;16:100082. doi: 10.1016/j.aicoj.2026.100082 (PMC13214333; doi:10.1016/j.aicoj.2026.100082)
Supplement: Supplementary file 1 [file mmc1.pdf]

Supplementary material:

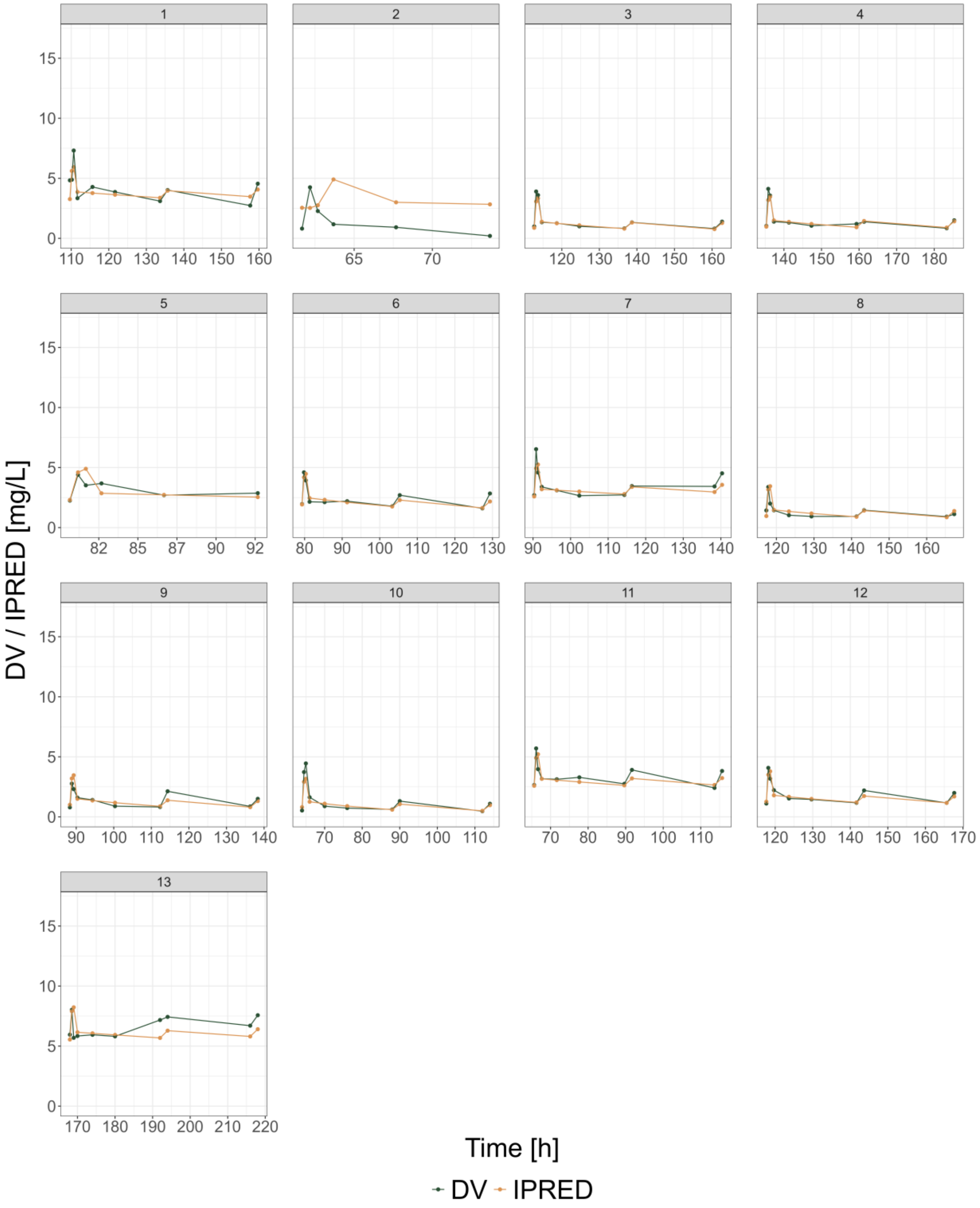

Figure S1: Measured pre-filter concentrations and individual model prediction

DV: dependent variable/measured pre-filter concentrations (in green), IPRED: individual model prediction (in orange)

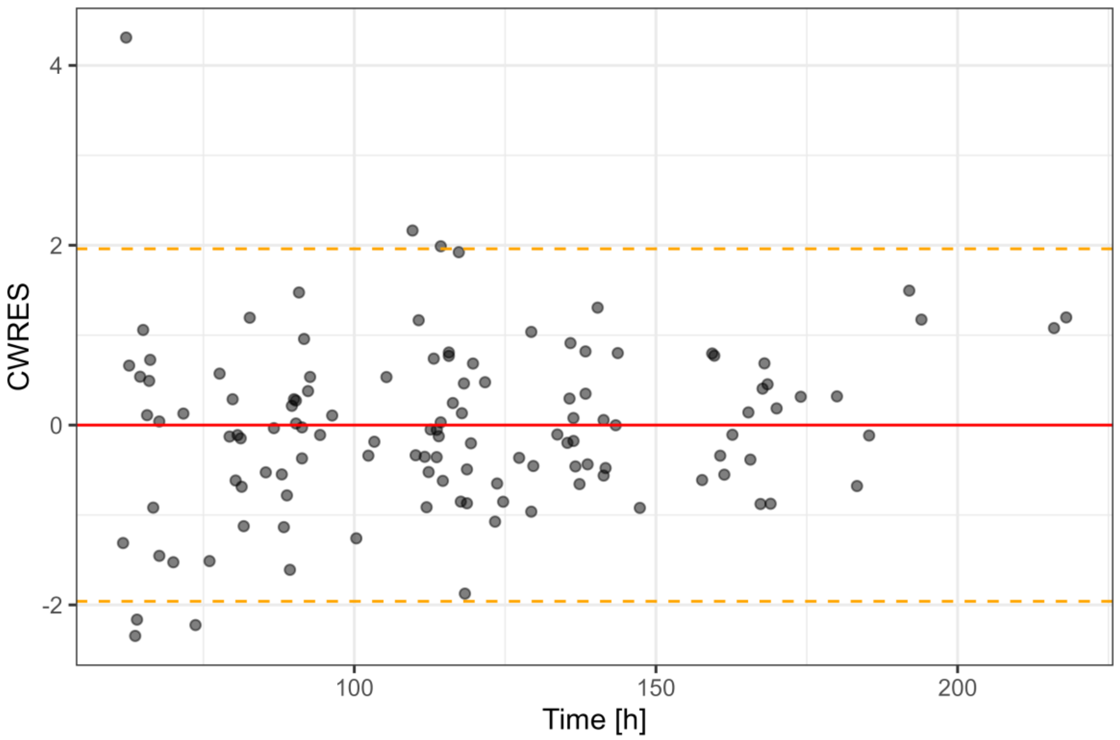

Figure S2: Conditional weighted residuals vs. time
CWRES: Conditional weighted residuals

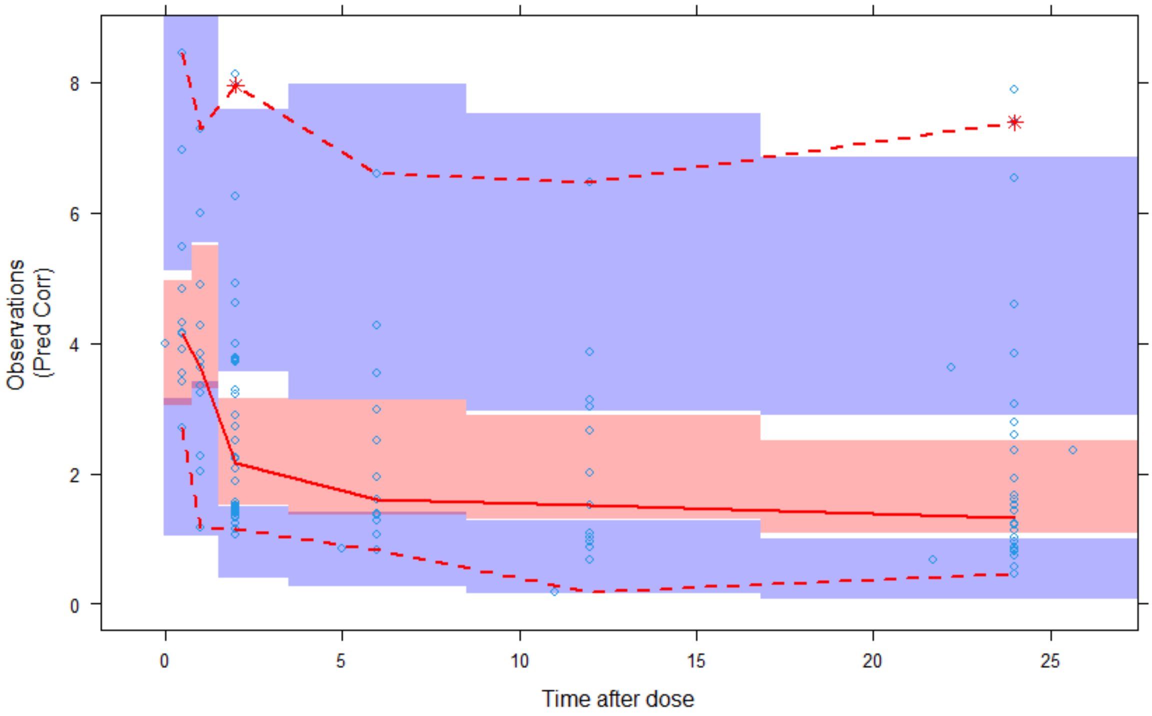

Figure S3: Prediction-corrected visual predictive check of isavuconazole concentrations versus time after dose
blue circles: prediction corrected observed plasma concentrations, solid red line: observed median, red field: model-based 95% confidence interval of median, dashed red lines: 5th and 95th percentiles of observed data, blue fields: model-based 95% confidence intervals of percentiles
